# Making Judgments of Learning (JOLs) for Others Produces Positive Reactivity

**DOI:** 10.3390/bs16091542

**Published:** 2026-09-01

**Authors:** Yunfeng Wei, Nicholas C. Soderstrom, Michelle L. Meade

**Affiliations:** Department of Psychology, Montana State University, P.O. Box 173440, Bozeman, MT 59717-3440, USA; wyunfeng@wustl.edu (Y.W.); mlmeade@montana.edu (M.L.M.)

**Keywords:** JOL reactivity, JOL for others, positive reactivity, cue utilization

## Abstract

The current study examined judgments of learning (JOL) reactivity in social contexts. Participants studied related and unrelated cue–target word pairs. Half of the participants were asked to predict the likelihood that other people would remember the cue–target pairs on a later memory test, whereas the other half were not. All participants then completed a final cued recall test. The results show that making JOLs for others, like making JOLs for oneself, produces positive reactivity. Specifically, making JOLs for others enhanced memory for related word pairs but not unrelated pairs, supporting the cue-strengthening hypothesis. This study serves as a starting point to examine the influence of making JOLs in social contexts and to understand the impact of cue utilization on JOL reactivity.

## 1. Introduction

Judgments of learning (JOLs) refer to individuals’ subjective evaluation of their learning (Dunlosky & Metcalfe, 2009; Rhodes, 2016, 2019). For example, when learning new information, individuals may think they will be more likely to later remember some parts of the new information relative to other parts. JOLs are important metacognitive assessments, because they inform strategies that individuals use to learn new information (e.g., spending more time restudying materials they feel they have not learned as well; Son & Kornell, 2008). Until recently, learning and JOLs were thought to be independent processes such that the act of making JOLs did not impact how well individuals learned the information, although Spellman and Bjork (1992) argued that these two processes are related. However, there is growing evidence of JOL reactivity: the finding that the mere making of JOLs impacts memory performance (for reviews, see Double et al., 2018; Ingendahl et al., 2025). JOL reactivity is a significant finding, but there are still many questions about the mechanisms underlying the effect and the boundary conditions of the effect (Ingendahl et al., 2025). In the current study, we examine JOL reactivity in social contexts. JOLs for others play a critical role in daily life. For example, teachers judge their students’ learning to tailor instructions for their students (Thiede et al., 2018, 2019; van de Pol & Oudman, 2024), and learning software and AI-assisted tutors help students with metacognitive processes by setting optimal goals and providing prompt feedback (Azevedo, 2007; Giannakos et al., 2025). Given the importance of JOLs for others, the current study examines whether JOL reactivity extends to making JOLs for others.

### 1.1. JOL Reactivity

Soderstrom et al. (2015) were one of the first to systematically examine JOL reactivity. They presented participants with cue–target pairs that were related and unrelated. Participants in the JOL condition studied the word pairs and estimated how likely they would be to recall the target on a later cued recall test. Participants in the no JOL condition studied the words without making a memory prediction. After three minutes, participants completed a cued recall test where they were presented with the cue and asked to type in the target associated with the cue. Soderstrom et al. found that making JOLs enhanced the memory performance for related word pairs compared to making no JOLs. This positive JOL reactivity was selective to related word pairs; there was no difference on the cued recall test for unrelated words with or without JOLs.

Soderstrom et al. (2015) explained their findings through the cue-strengthening hypothesis, which states that making JOLs strengthens individuals’ utilization of cues that inform JOLs. According to Koriat (1997), learners use three types of cues to judge their learning: intrinsic cues (characteristics of to-be-learned materials), extrinsic cues (learning conditions), and mnemonic cues (mnemonic indicators). Further, intrinsic cues and extrinsic cues can be utilized in two distinctive ways: a direct theory/belief-based and an indirect experience-based way through mnemonic cues. For example, students may make higher JOLs for related word pairs because (1) they believe related words are easier compared to unrelated pairs (belief-based), and/or (2) mnemonic indicators (e.g., processing fluency) show related word pairs may be better remembered. Therefore, the cue-strengthening hypothesis suggests that making JOLs strengthens the processing of relatedness (an intrinsic cue) and produces superior memory performance. This positive reactivity (i.e., making JOLs enhances memory) has been supported by a substantial amount of research (e.g., Chang & Brainerd, 2023; Janes et al., 2018; Kaya & Mulligan, 2026; Maxwell & Huff, 2022, 2023, 2024; Rivers et al., 2021; Yavuz & Besken, 2026; Zheng et al., 2025; for meta-analysis, see Double et al., 2018; Ingendahl et al., 2025; cf. Ingendahl & Undorf, 2024; Witherby et al., 2026).

Importantly, however, there is also evidence that JOLs impair memory performance (negative reactivity, e.g., Halamish & Undorf, 2020; Ingendahl & Undorf, 2026; Mitchum et al., 2016; Zhao et al., 2023). In a study by Mitchum et al., the authors used word pairs with varying levels of relatedness and found that making JOLs did not enhance memory for related items but impaired memory for unrelated items. They proposed two hypotheses to explain the negative reactivity: (1) the dual task hypothesis that making JOLs requires cognitive resources and interferes with learning unrelated or difficult materials; (2) the changed goal hypothesis that making JOLs helps individuals shift their learning goal from mastering all items to remembering easy-to-learn items, which should produce positive reactivity for related word pairs and negative reactivity for unrelated ones.

Considered together, there is strong evidence that making JOLs influences memory. There is also growing interest in understanding the boundary conditions of the effect and the different factors that inform theoretical mechanisms. As noted above, one factor seems to be related vs. unrelated pairs, such that JOLs produce positive reactivity for related pairs and negative reactivity for unrelated ones. In addition, Ingendahl et al. (2025) showed that JOL reactivity is influenced by various factors, including type of test (cued recall vs. recognition), language, and baseline memory performance. The current experiment further informs the boundary conditions of JOL reactivity by examining whether JOL reactivity effects extend to making JOLs for others.

### 1.2. JOL Reactivity for Others

Making JOLs for others is different from making JOLs for oneself, but individuals may process the cues comparably. Wei et al. (2026) proposed a framework adapted from Koriat’s (1997) cue utilization framework to better explain how cues are utilized when making JOLs for others. Specifically, Wei et al. claimed that individuals not only consider intrinsic, extrinsic, and mnemonic cues but also incorporate social cues to inform JOLs for others. For example, teachers consider students’ background information, such as personality, parents’ attitudes, and environment, when making JOLs (for a review, see Thiede et al., 2019). Furthermore, Wei et al. differentiated between mnemonic cues for judges and learners. That is, learners’ mnemonic cues, such as past memory performance on a similar task, can be utilized by judges to inform JOLs for learners through judges’ beliefs. In addition, judges may interpret cues differently according to their prior learning experiences. Therefore, when individuals have access to a cue (e.g., relatedness), they may use the cue according to their beliefs (“related cues should be easier for others”) and their experiences (“I learned related words better than unrelated words”).

The argument that making JOLs for others requires utilization of both cues and judges’ experiences has been supported by previous studies. For example, Tullis (2018) asked participants to estimate others’ performance on trivial questions before or after answering those questions themselves and found that the order of estimation and answering questions made a difference in participants’ estimation. In addition, Koriat and Ackerman (2010) asked their participants to make JOLs for another learner before or after they learned the same materials and discovered that the judges’ learning experience changed how they interpreted the study time of the learner (an extrinsic cue) (see also, Undorf & Erdfelder, 2011). Therefore, there is converging evidence that making JOLs for others prompts judges to process both cues and subjective learning experiences, which is consistent with Koriat’s (1997) framework that intrinsic and extrinsic cues are utilized through both theories and experience.

However, it is worth noting that individuals may adjust the extent of utilizing cues through beliefs and experiences when making JOLs for others compared to making JOLs for oneself. Specifically, previous studies showed that individuals generate memory cues for others more based on associative strength (theories) between target words and cues, but they rely more on personal and idiosyncratic information (experiences) when generating memory cues for themselves (Harris & McIlwain, 2026; Tullis & Benjamin, 2015; Tullis & Finley, 2021) and long-term partners (Harris & McIlwain, 2026).

To summarize, individuals utilize cues (e.g., relatedness) both through their beliefs and experience when making JOLs for themselves and others. However, there is a discrepancy between these two processes. Specifically, when processing memory and learning for oneself, individuals tend to utilize their experience and personal information more, but when processing others’ learning, they tend to consider more general information and their beliefs. Given that making JOLs for oneself and others have similarities and distinctions, it is necessary to know whether JOL reactivity findings can be replicated when making JOLs for others. Theoretically, this study could help researchers better understand the relationship between JOL reactivity and the manner in which cues are processed.

### 1.3. The Present Study

We examined whether JOL reactivity effects extend to making JOLs for others. Previous frameworks (Koriat, 1997; Wei et al., 2026) indicated that making JOLs for others involves processing both cues and learning experiences, yet it is unknown whether making judgments about others produces JOL reactivity. There were three hypotheses regarding the findings in the current study according to previous accounts. (1) We would replicate positive JOL reactivity for related word pairs when participants made JOLs for others and no JOL reactivity for unrelated word pairs when participants made JOLs for others (the cue-strengthening account). (2) Participants would show negative JOL reactivity for unrelated word pairs because they do not have adequate cognitive resources to study difficult materials and judge others’ learning simultaneously (the dual-task hypothesis). (3) Participants would show positive reactivity for related pairs and negative reactivity for unrelated pairs after making JOLs for others because judging others’ learning may make relatedness more saliant, and participants would shift their goal to master more related items and put less effort in mastering unrelated items (the changed-goal hypothesis).

## 2. Methods

### 2.1. Design

The experiment used a 2 × 2 mixed factorial design. The first independent variable in the current study was relatedness between word pairs (related vs. unrelated) manipulated within subjects, and the second independent variable was JOLs (making JOLs or not) manipulated between subjects. The dependent variable was participants’ memory performance on the final cued recall test.

### 2.2. Participants

Soderstrom et al. (2015) showed that the effect size for JOL reactivity (i.e., the interaction between making JOLs and relatedness of word pairs) was *η*^2^ = 0.13 (Cohen’s *f* = 0.387, Kim, 2016). However, this effect size was for making JOLs for oneself. To be conservative, we cut the effect size by one third to better investigate the JOL reactivity for making JOLs for others by setting Cohen’s *f* = 0.25, Power = 0.90 in G*Power (version 3.1.9.7, Faul et al., 2007), and the power analysis indicated a minimum sample size of 46 participants. We recruited 79 participants in total from an online SONA subject pool, but three participants were excluded from data analysis for failing to follow instructions. Thus, a final sample of 76 undergraduate students (38 in the JOL condition and 38 in the no-JOL condition) was analyzed, with a mean age = 19.15 years (*SD* = 3.09). The gender distribution was 46.05% male, 50% female, and 3.95% non-binary/other. Most of the participants (98.68%) were White. All participants received course credit as compensation.

### 2.3. Materials

We used the cue–target word pairs from Soderstrom et al. (2015). Half of the pairs were highly related (e.g., Flight—Airplane), whereas the other half of the pairs were unrelated (e.g., Fruit—End). In total, there were 30 related word pairs and 30 unrelated word pairs. We also used the cued recall test from Soderstrom et al. (2015), which included all 60 cue words. Additional materials included a locally developed demographics questionnaire.

### 2.4. Procedure

Experiment construction and data collection were completed through the Gorilla Experiment Builder (www.gorilla.sc, accessed on 4 March 2026, Anwyl-Irvine et al., 2020) following the paradigm in the previous study (Soderstrom et al., 2015). After consenting to participate in the experiment, participants read the instructions on their computer screen. Specifically, they were asked to study word pairs and were instructed that they would be later tested on the word pairs—their job would be to recall the target word when the cue word was provided. Word pairs were presented one at a time for eight seconds each. Participants in the no-JOL condition were only told to study each pair for eight seconds. Participants in the JOL condition were told to study word pairs and predict the chances that other people would remember the word pair halfway through studying each pair (after four seconds). They entered in their numeric estimate of others’ recall using a scale from 0–100, where 0 indicates “not likely at all”, and 100 indicates “absolutely likely”.

Two related and two unrelated practice word pairs were provided first for participants to become familiar with the task. Then, participants proceeded to study all word pairs. Once participants studied all word pairs, they were provided with a short filler task in which they were instructed to type as many U.S. states as possible in three minutes.

For the memory task, participants were presented with all 60 cue words one at a time. For each cue presented, they were instructed to type in the corresponding target word within eight seconds. The test timing was computer based such that no matter how quickly participants provided their responses, the entire eight seconds elapsed before the screen advanced to the next cue word. After the memory task, participants completed a short survey to gather their demographic information.

## 3. Results

### 3.1. JOLs

On average, participants in the JOL condition provided their estimation for 57.13 (*SD* = 3.83) out of 60 word pairs (95.22%). A paired-sample *t* test demonstrated that participants made higher JOLs for related word pairs (*M* = 74.58, *SD* = 14.22) compared to unrelated pairs (*M* = 19.34, *SD* = 9.23), *t*(37) = 23.76, *p* < 0.001, Cohen’s *d* = 3.86, suggesting that participants used relatedness as a cue, believing that others would remember related pairs better than unrelated pairs (Harris & McIlwain, 2026; Tullis & Benjamin, 2015; Tullis & Finley, 2021).

### 3.2. Memory

**Figure 1:**
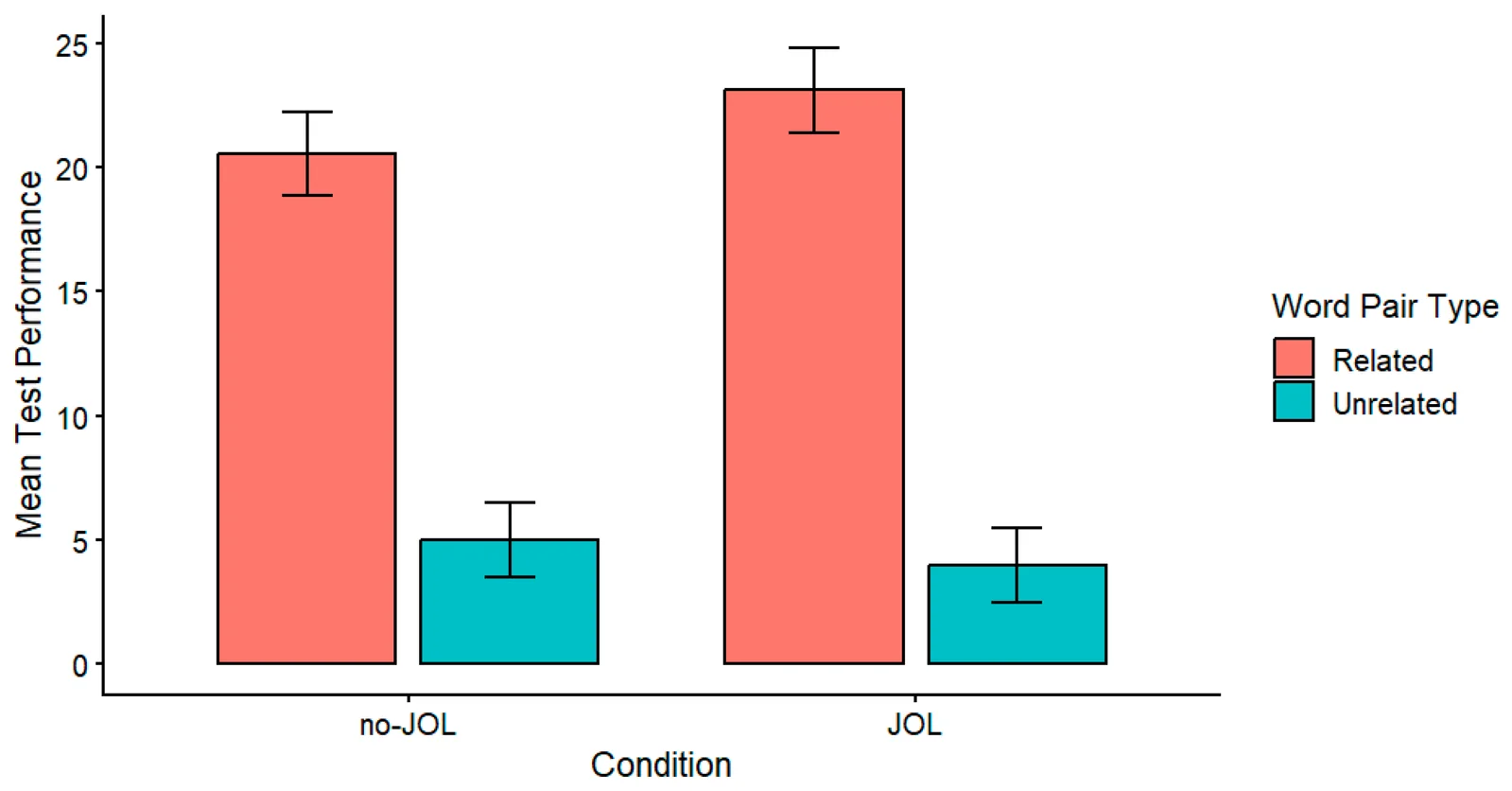
Number of items recalled as a function of JOLs and relatedness. Error bars represent 95% confidence intervals.

We conducted a 2 (Relatedness: related vs. unrelated) × 2 (JOL: JOL vs. no-JOL) mixed ANOVA to examine the proportion of target words correctly recalled on the cued recall test. As shown in Figure 1, the ANOVA revealed a significant main effect of Relatedness, *F*(1, 74) = 1209.14, *p* < 0.001, *η*^2^*_p_* = 0.94, revealing that participants recalled more related target words (*M* = 21.80, *SD* = 5.41) relative to unrelated target words (*M* = 4.47, *SD* = 4.61). The main effect of JOLs was not significant, *F*(1, 74) = 0.56, *p* = 0.46, *η*^2^*_p_* = 0.007. Importantly, there was a significant interaction between Relatedness and JOLs, *F*(1, 74) = 13.22, *p* < 0.001, *η*^2^*_p_* = 0.15. Bonferroni corrected pairwise *t*-tests showed that, for related pairs, participants remembered more related target words when making JOLs compared to making no JOLs, *t*(74) = 2.13, *p* = 0.04, Cohen’s *d* = 0.50, thus demonstrating a positive JOL reactivity effect. However, making JOLs did not affect the performance for unrelated word pairs, *t*(74) = 1.00, *p* = 0.32, Cohen’s *d* = 0.23.

## 4. Discussion

The current study was the first to extend JOL reactivity effects to making JOLs for others. The participants studied both related and unrelated word pairs and made JOLs on half of the items. We found positive JOL reactivity effects for related word pairs and no JOL reactivity effects for unrelated word pairs. These findings show that JOL reactivity effects indeed generalize to making JOLs for others and offer a starting point for understanding how social context influences JOL reactivity.

The finding that making JOLs for others enhanced memory performance for related word pairs relative to making no JOLs is consistent with previous findings (e.g., Maxwell & Huff, 2022, 2023; Soderstrom et al., 2015; for meta-analysis, see Double et al., 2018; Ingendahl et al., 2025). Importantly, this effect was selective to related word pairs; making JOLs did not influence memory performance for unrelated word pairs. The null JOL reactivity effects in the current study are consistent with some previous studies (e.g., Soderstrom et al., 2015) but inconsistent with other studies showing negative reactivity for unrelated or difficult items (e.g., Halamish & Undorf, 2020; Ingendahl & Undorf, 2026; Mitchum et al., 2016; Zhao et al., 2023). The inconsistent results can be explained by the differences in initial acquisition type and time allocation (self-paced acquisition, Mitchum et al., 2016), the language used in the experiments (German, Ingendahl & Undorf, 2026), and the type of tasks (Order reconstruction, Zhao et al., 2023). Nonetheless, the current findings do not align with the dual task hypothesis or the changed goal hypothesis of JOL reactivity because we did not find negative reactivity. Instead, the findings in the current study support positive JOL reactivity and the cue-strengthening hypothesis (Soderstrom et al., 2015) by revealing a significant improvement in memory performance for related word pairs but no effect for unrelated pairs. An informative comparison can be made between the current results and the literature on pre-study JOLs. Pre-study JOLs are made before learners have any experience with the materials and are therefore assumed to rely largely on beliefs about learning rather than on mnemonic experience. Notably, prior work has found little evidence of reactive effects following pre-study JOLs (Li et al., 2024), a finding that aligns with the cue-strengthening account of JOL reactivity (i.e., a search for diagnostic cues at the time JOLs are made) because relatively few item-specific cues are available at the time of judgment. By comparison, participants in the present study made JOLs for others while viewing the word pairs themselves. Although these judgments are likely influenced by beliefs about how others learn, participants also had access to the study materials and their own subjective impressions during encoding. Consequently, JOLs for others may not be directly comparable to pre-study JOLs and may instead involve processes that overlap more closely with JOLs for oneself. From a theoretical standpoint, this distinction is important because it is more consistent with the cue-strengthening account of JOL reactivity than other accounts.

We attempted to generalize previous findings to JOLs for others, leading to more research on the effects of making JOLs for others in social contexts. By extending JOL reactivity effects to making JOLs for others, the current study can inform the mechanisms underlying JOL reactivity. Koriat’s (1997) cue utilization framework shows that intrinsic and extrinsic cues can be utilized in both theory/belief-based and experience-based ways when making JOLs for oneself. Wei et al. (2026) claimed that these cues can also be used in both direct theory/belief-based and indirect experience-based ways when judging other people’s learning. Despite these similarities, predicting ones’ own learning relies heavily on idiosyncratic and personal information, whereas predicting others’ learning seems more dependent on general associative strength between cues and target words (Harris & McIlwain, 2026; Tullis & Benjamin, 2015; Tullis & Finley, 2021) Taken together, even though making JOLs for others is different from making JOLs for oneself in terms of the extent of processing cues through beliefs and/or experience, the present study suggests that making JOLs for others, like making JOLs for oneself, can produce JOL reactivity. Future research is needed to tease apart these two processes—utilizing cues through beliefs versus experiences—to better understand the effects of each process on JOL reactivity.

The limitations of the current study help motivate future research. One limitation is that even though students benefit from making JOLs when learning related items, it is still unknown how different pathways of utilizing cues affect reactivity. Specifically, individuals may benefit from JOL reactivity because they access their own learning experience, or merely utilize cues according to their beliefs, or both. Future research is required to understand the effects of different methods of utilizing cues. It is also necessary to replicate findings in more realistic settings. For example, can students benefit from judging their peers’ learning in small groups? Further, various materials should be used in future research to generalize the results beyond word pairs and to examine the influence of other cues on JOL reactivity. Lastly, we did not include a condition in which participants made JOLs for themselves. Future research might directly examine JOL reactivity for oneself versus others.

## 5. Conclusions

The present study generalizes prior JOL reactivity findings to making JOLs for others by demonstrating that making JOLs for others enhances judges’ memory performance for related word pairs. This represents a starting point for understanding the effects of making JOLs for others in shaping memory in realistic social contexts, because judging others’ learning plays a critical role in interactive learning environments.

## Data Availability

The data presented in this study are openly available in the Open Science Framework (https://osf.io/75muj/files/osfstorage, accessed on 23 June 2026).
